# A Practical Treatise on the Diseases of the Skin, Arranged with a View to Their Constitutional Causes and Local Characters, &c.

**Published:** 1837-07

**Authors:** 


### Art. XIII.

1. *A Practical Treatise on the Diseases of the Skin, arranged with a View to their Constitutional Causes and Local Characters, &c.* By Samuel Plumbe, Surgeon to the St. Giles' and St. George's Parochial Infirmary, &c. *Fourth Edition.—London,* 1837. 8vo. pp. 607; four Plates.
2. *A Practical Compendium of the Diseases of the Skin, including a particular Consideration of the more frequent and intractable Forms of these Affections; with Cases.* By Jonathan Green, M.D. &c. *Second Edition.—London,* 1837. 8vo. pp. 371; two Plates.

It is a curious anomaly that an order of diseases, presenting, from their situation, peculiar facilities for observation, and for the development of a correct pathology, should be so involved in obscurity as diseases of the skin undoubtedly are; that their arrangement upon really scientific principles should be, even at this day, an object unattained; and the treatment of them, in most of its parts, little better than mere empiricism. Notwithstanding the labours of many distinguished and diligent enquirers, both in this country and abroad, the system of arrangement of Drs. Willan and Bateman, founded upon the divisions of Plenck, confessedly artificial as it is in principle, still remains, with some modifications and additions, the recognized guide in the acquirement of a knowledge of these affections; while the following passage, from the Introduction to Dr. Green's treatise, is but too just an account of the empirical and unsatisfactory mode of treatment hitherto almost universally followed.

"All that has been done," he observes, "in regard to the treatment of this class of complaints in this country, of late years, amounts to a few experimental trials of certain heroic remedies, (among which mercury, arsenic, and prussic acid, figure in the foremost rank,) and endeavours to force ourselves into the belief that cutaneous diseases were uniformly owing to some mysterious and indefinable affection of the digestive organs, nowise observable, in nine cases out of ten, in any derangement of their functions, but for which the blue or Plummer's pill, and purgative medicines, were the approved specifics. One or other of these pills was, therefore, almost uniformly commenced forthwith, and ample doses of purgative medicine were prescribed. This course being persevered in, and no good resulting, as was most frequently, though perhaps by no means invariably, the case, small doses of the hydrargyri oxymurias, combined with decoction of sarsaparilla, followed. This failing in like manner, Fowler's arsenical solution, and perhaps decoction of dulcamara, were next recommended; and these, either proving ineffectual against the disease, or causing some suspicious or unpleasant disorder of the system, were in their turn abandoned. The patience of the physician as well as of the patient being now worn out, they usually parted company at last, little satisfied with each other; the one lamenting the obstinacy of skin-complaints, the other inveighing against the inefficacy of medicine, if not against the ignorance of its practitioners." (P. 2.)

Dr. Green thinks that more rational views are entertained, and more rational practice has been introduced, by the French physicians, more especially in the employment of various modifications of the bath and the douche; but, at the same time that we are willing to admit the utility of baths of different descriptions, and their beneficial effects in regulating the functions of the skin and its appendages, we much fear that the indiscriminate mode in which these measures are had recourse to by French practitioners, at least in the Parisian hospitals, is only an additional exemplification of the correctness of the opinions expressed by Dr. Green with reference to preceding modes of treatment. In fact, as Mr. Plumbe justly remarks, the descriptions of the French writers (and we can bear testimony to their general accuracy,) shew that, whether from neglect, from a meager and impoverished diet, from constitutional peculiarity, or other cause, the instances of cutaneous disease met with in France are frequently such as in this country we can scarcely appreciate; and yet the use of baths in Paris, and other parts of the continent, is almost universal among the poor as well as the more elevated ranks, every facility being provided for their supply.

It is to other causes, then, as well as to mechanical obstruction of the functions of the skin, or to the accumulation of irritating secretions upon its surface, that we must look for a reason of the prevalence and severity of skin disease; and, however beneficial the use of baths, medicated or simple, or of the douche, and other topical applications, may be in certain forms of these affections, the reproach of pure empiricism will not be taken away until more just and more comprehensive views of pathology, and views more accordant with general principles, are impressed upon the medical practitioner.

Without pledging ourselves to the approval of the principles of arrangement adopted by Mr. Plumbe, in the present and preceding editions of his treatise, (which, as it appears to us, are much too limited in their application for general use,) we cannot but admit that the attempt is at least a step towards the attainment of a more correct pathology. More extended enquiry is however necessary, and a closer investigation than has yet taken place, instituted, with this express object, into the nature of many of the forms of cutaneous disease, is requisite, before these or any other principles can be received as a general guide to the arrangement and treatment of this class of affections. Mr. Plumbe establishes six sectional divisions, which he characterizes as follows:— 1. Diseases which obtain their distinguishing characteristics from, or originate in, local peculiarities of the skin; 2. Diseases chiefly marked by chronic inflammation of the vessels secreting the cuticle, producing morbid growth of this structure, and generally dependent on debility of system; 3. Diseases exerting a probably salutary influence on the system, originally produced by, and usually symptomatic of, deranged digestive organs, and characterized by active inflammation; 4. Diseases of a mixed character, essentially dependent on active inflammation, with which the constitution is not necessarily connected; 5. Diseases dependent on debilitated and deranged states of system, and consequent diminished tone of the vessels of the cutis; 6. Fungoid diseases of the skin.—Under the first of these sections are arranged Acne, Scrofulous Inflammation and Ulceration of the Follicles, Sycosis, Lupus, and Porrigo;—the Second includes Lepra, Psoriasis and Pellagra, and Pityriasis;—the Third, Porrigo favosa, Strophulus, Lichen and Prurigo, Urticaria, Herpes, and Furunculus or Boils;—the Fourth, Impetigo, Scabies, and Eczema;—the Fifth, Purpura, Aphtha, Pompholyx and Pemphigus, Ecthyma and Rupia, and Erythema nodosum;—and to the Sixth are referred Ichthyosis, and Warts. Under the head of what are termed "Concluding Remarks," we find an allusion to Erythema and an account of Roseola, chiefly extracted from M. Rayer's work;—and there is an Appendix, containing a series of short dissertations upon Horny Productions of the Cutis, Corns, &c.; Parasitic Insects; Elephantiasis, Barbadoes Leg, Pedarthorace, Hypertrophy of the Scrotum, &c.; Aleppo Pustule, &c.; Leprosy of the Danes, of the Cossacks, of the inhabitants of India, and of the Jews; Beriberi; Sibbens; Frambœsia, &c.

An examination of the preceding list at once shews that the author has entirely failed in producing a natural arrangement, even of those affections of the skin which he has thrown together in his sectional divisions, without taking account of the omissions which he has attempted to remedy in his "Concluding Remarks" and Appendix. Not only, therefore, does this treatise labour under the very important objection of giving a most imperfect account of numerous affections which ought to find a place in a work professing to be a monograph of "the Diseases of the Skin;" but, as a nosological arrangement, and even as a guide to the inexperienced practitioner, it is comparatively useless. Considered as a system of arrangement, it can only be viewed in the light of a series of separate essays, in which the attempt has been made to develope certain natural features of resemblance. The attempt is laudable, and deserves every encouragement; it may in some few instances be thought to have partially succeeded; but we much fear that the principles upon which the sectional divisions are founded are not such as are of general application, and that, independent of an obscurity and undefined character which the sections present, capable perhaps of being removed or materially remedied by further investigation, they must fail both in affording a natural arrangement of these diseases, and in facilitating the acquirement of knowledge respecting them.

Dr. Green follows the arrangement of Willan and Bateman, with some modifications, perhaps improvements, which are in part derived from the elaborate treatise of M. Rayer. We recommend to Dr. Green, as well as to Mr. Plumbe, as a guide to their future researches, the valuable observations of MM. Breschet and Roussel de Vauzème on the structure of the skin, of which we have given a pretty full account in a preceding Number. Mr. Plumbe, indeed, refers to these investigations in his preliminary remarks, although the abstract which he makes of them is not so full and clear as could be wished, considering their important bearing upon the morbid conditions of this texture and its appendages. Those who have not the opportunity of referring to the original memoirs may consult with advantage the notice of them and the accompanying plates, in our second volume.

The introductory remarks in the work of Mr. Plumbe are, in general, judicious; but we must protest against the dangerous tendency of such observations as are contained in the following extract, especially as the young and inexperienced practitioner is so likely to be misled by them.

"The prejudice is of old standing," says the author, "that cutaneous diseases ought not to be suddenly removed, and many and long are the tales recorded of vital mischief following their sudden disappearance. To combat that prejudice successfully is not to be expected by any author, but something may yet be alleged in the shape of argument and fact, calculated to diminish or modify it. There can be no denial of the powerful influence of irritation, inflammation, vesication, or ulceration of the cutaneous surface, in warding off the mischief and counteracting the progress of internal disease. A practice founded on this principle is universally adopted by medical men, and very frequently with the greatest success, where manifest symptoms of internal disease exist. But the question naturally occurs, how, if there be no signs of such internal disease, can the practitioner be afraid of curing his patient, as speedily as possible, of the external affection? The truth is, that few or no diseases of the surface require any but (to use a phrase not common in medicine,) *off-hand* treatment, unless there be reason to apprehend that internal organic disease really exists or is threatened. It is seldom, indeed, under such circumstances, that diseases of the skin are of more than secondary importance; and hence the general position I have endeavoured to lay down, of considering them in all their parts and characteristics in conjunction with the state of constitution of the patient. When, therefore, there is no reason to suspect the integrity of any vital organ, I apprehend nothing in the form of mischief may be expected if the skin disease be got rid of at once, whether by external applications or internal medicines. The doctrine of the humoral pathology has supported, and still continues to support, a contrary notion, but I believe it to be entirely fallacious; and I have never found cause to regret having acted on that opinion." (P. 34.)

We much fear that, should the young practitioner, relying on the absence of external indications of internal disease, in cases of long-continued cutaneous affection, attempt to cure such affection in the *off-hand* manner recommended by Mr. Plumbe, he will have very serious cause to regret having acted on that opinion. It is an idle assertion to make, that "many and long are the tales recorded of vital mischief" following the sudden disappearance of cutaneous disease, without at the same time informing the reader by whom these many and long tales have been recorded. When it is understood that facts, related by such men as Alibert, Philippe Boyer, Bouchard, Campet, Chaussier, Esquirol, Hoffmann, Klein, Lorry, Petit, Portal, Raymond, Schenk, &c., to say nothing of many eminent writers in our own country, are to be found bearing testimony to the reality of this vital mischief, it will, we think, require more in the shape of fact and of argument to abate the so-called prejudice of not too hastily attempting to get rid of cutaneous disease than Mr. Plumbe has yet brought forward.

But Mr. Plumbe establishes one important section of these diseases upon the principle, and, as it appears to us, the correct principle, although perhaps scarcely available for his purpose, that the diseases referred to it are usually symptomatic of a deranged state of the digestive organs; and it is only necessary to collate the leading characters given of each of the six sectional divisions into which he has distributed skin diseases, to observe that, in the diseases included in four of these divisions, Mr. Plumbe himself considers the system generally to be materially implicated. Surely, then, to say that "few or no diseases of the surface require any but *off-hand* treatment," is much too off-hand an assertion; and the qualification which immediately follows, by way of exception, should be regarded rather as the general rule. We should rather say that, as, in the greater number of cases, there is reason to apprehend that internal organic or functional disease really exists, or is threatened, disease of the surface by no means admits of *off-hand* treatment; and we should say further, that, even in cases of scabies,—a disease which is now known, from the researches of M. Raspail, to take its rise from the presence of a parasitic insect,—when the affection has been of long standing, it behoves the physician to investigate carefully the secondary effects which may have resulted upon the constitution of the patient, and to be prepared to appreciate and to remedy any injurious consequences which have arisen in the course of the disease, or which may arise in the progress of cure.

These views are in accordance with those of the most judicious practitioners, and with the best authorities upon the subject. "The more we study," says M. Rayer, "the development and tendency of the greater number of the diseases of the skin which occur independently of any outward appreciable cause, the more we become convinced of their connexion with the state of the constitution, and of the necessity there is for considering them under this point of view before we think of undertaking their cure, or even of interfering with them so as to modify their progress." M. Alibert has expressed the same opinions; while Lorry, Esquirol, and many other eminent writers, abound with cases illustrative of the mischief which too often arises from a disregard of them.

Dr. Green's treatise seems to have been written chiefly with the view of bringing forward his experience in the employment of the various modifications of the bath. The utility of these, as adjuncts, we are not disposed to question, although the author appears much to overrate their value in ascribing to them effects which are probably due, in part at least, to the employment of constitutional measures previously or conjointly adopted. His remarks on the general treatment are judicious, although not possessed of much novelty. Mercurials and antimonials he considers as being greatly overrated; the latter possessing very little effect in the cure of skin disease, and the former being frequently positively injurious, which, under the indiscriminate employment too often made of this mineral, is but too likely to be the case. In those forms of chronic cutaneous disease in which the eruption is attended with an inflamed state of the cutis, we can bear decisive testimony to the efficacy of depletory measures, general or local; general, by means of venesection, and reduced or regulated diet, when the constitution will admit of it; and local, by means of leeches and emollient fomentations applied close to or upon the affected parts. Some of the varieties of porrigo we have seen especially benefited by repeated applications of leeches to the scalp, after the hair had been cut close, and the crusts carefully removed.

A great variety of remedies have been employed in the treatment of different forms of skin disease, many upon no better principle apparently than mere experimental caprice; and of these not a few have been highly extolled as capable of curing the most obstinate of these affections. If, however, we take the trouble to collate the several observations brought forward by various authors in favour of certain remedies with the experience of others, we shall find that those only have retained a place in the practice of the unbiassed and intelligent physician, which may be shewn to act in accordance with some general or recognized principles: for instance, in the syphilides, or the various forms of papular, squamous, tubercular, or other eruption consequent upon a syphilitic infection, mercurials, sarsaparilla, and nitric acid; in erysipelas, tartarized antimony; iodine in the scrofulous forms, or in those occurring in scrofulous constitutions; the different preparations of iron in such as are accompanied with, or have been preceded by, irregularities in the catamenial functions; tonics internally, and stimulant topical applications, where there is evidence of constitutional or local debility; general and topical bleeding, with aperients and regulated diet, in the more inflammatory states; and lastly, alteratives, alkalines, or acids, in those forms which are connected with, or dependent upon, a disordered state of the digestive functions.

Both the treatises before us afford abundant evidence in confirmation of these remarks. The frequent benefit derived from copious ablutions, and from the baths and sulphur fumigations so strongly recommended by Dr. Green, may be resolved into the same principle; for the cases in which these remedies are found most efficacious are precisely those in which the functions of the skin generally are deranged.

In the observations which we have been induced to make upon the works of Dr. Green and Mr. Plumbe, we have not felt ourselves called upon to enter into an extended analysis of their contents, since both treatises have been already some time before the profession, while the consideration of the several subjects in detail would have encroached too much upon our limits. For these we must refer to the works themselves, in which our readers will find them in general judiciously treated. Dr. Green's Compendium is decidedly the most complete, and is further valuable as containing the results of his own experience in the treatment of the cases, with the advantage of well-regulated baths and fumigations. In many varieties of skin disease, more especially in the more chronic forms, the evidence of the benefit derived from such applications is decisive: at the same time we must observe, that the employment of the sulphur-fumes in the more acute states, even from Dr. Green's own statements, seems very questionable; and, when we find this remedy extolled in erysipelas and acute erythema, we are induced to hesitate in receiving the opinions of the author with that confidence which we should otherwise have been disposed to give. In the cases detailed of the latter disease, we much question whether the sulphur-fumes were not decidedly injurious; and there can be little doubt that the cure of the affection is to be attributed rather to the judicious employment of venesection and other appropriate remedies. The same observations apply equally to the acute stages of prurigo, lepra, &c., in which no benefit seems to have been derived from the fumigations until the inflamed state of the skin was subdued, and the eruption materially abated, by the use of depletory measures. The *modus operandi* of the sulphur-fumes seems to consist in the inducing of a process of desquamation, by which the diseased and thickened cuticle is extensively removed, permitting the formation of a new cuticle upon the surface of the skin, restored by this and other remedies to a more healthy state.

Mr. Plumbe's Treatise, though labouring under the disadvantages which we have before pointed out, has yet the merit of presenting some of the subjects which it embraces in connexion with others to which they are nearly allied, and which had been kept too far apart in the writings of preceding authors. It has also the merit of simplifying considerably, and reducing to their just rank, the minor divisions into genera and species of Willan, Bateman, and Alibert, and of presenting some important views in respect of treatment which, with the precautions previously pointed out, and which indeed the author himself occasionally enforces, are deserving the attention of the practitioner.

We must, however, before concluding, so far depart from our intention of not touching upon the consideration of particular subjects, as to recommend for careful perusal the chapter in Mr. Plumbe's work on Scrofulous Inflammation and Ulceration of the Follicles. The observations there given present, what we believe to be, a faithful picture of the destructive ravages which follicular cutaneous disease sometimes occasions in scrofulous constitutions. It is clear that no local measures could avail under the condition here so forcibly described; the only treatment which, as it seems to us, can be of any real benefit, is a removal from low, damp, or crowded and ill-ventilated situations to a mountainous or maritime district, regulated and nutritious diet, and perhaps the exhibition of iodine internally.

---

